# Changes in health-related quality of life: a longitudinal registry study among over 15,000 participants of a digital education and exercise therapy for knee and hip osteoarthritis using EQ-5D-5L

**DOI:** 10.1186/s12955-026-02634-5

**Published:** 2026-09-19

**Authors:** Ali Kiadaliri, Andrea Dell’Isola, Armaghan Mahmoudian, Leif E Dahlberg, L Stefan Lohmander

**Affiliations:** 1 Department of Clinical Sciences Lund, Orthopedics, Lund University, Remissgatan 4, SE-221 85, Lund, Sweden; 2 Division of Insurance Medicine, Department of Clinical Neuroscience, Karolinska Institutet, Stockholm, Sweden; 3 Department of Movement Sciences and Health, University of West Florida, Pensacola, USA; 4 Arthro Therapeutics, Malmö, Sweden

**Keywords:** Digital care, Education and exercise, EQ-5D-5L, Health-related quality of life, Osteoarthritis, Sweden

## Abstract

**Objective:**

To explore changes in health-related quality of life (HRQoL) measured using EQ-5D-5L among participants of a digital education and exercise therapy for knee and hip osteoarthritis (OA).

**Methods:**

A descriptive longitudinal registry-based study among participants aged 40 + years enrolled in the digital program with responses to EQ-5D-5L at enrolment and at least one follow-up at 3- [*n* = 15147] or 12-months [*n* = 4586]. Changes in EQ-5D-5L profile from enrolment were examined using the Paretian classification of health change (PCHC).

**Results:**

At enrolment, selfcare and pain/discomfort were the EQ-5D-5L dimensions with the least and most reported problems, respectively. The percentages of participants reporting improvements in EQ-5D-5L dimensions ranged from 12.9% (14.0%) for selfcare to 33.0% (38.4%) for pain/discomfort at 3 (12) months follow-ups. The corresponding percentages in reporting worsening ranged from 8.0% for selfcare to 15.4% for usual activities at 3 months and from 10.0% for selfcare to 16.4% for mobility at 12 months. At all timepoints, “slight problems” in the pain/discomfort dimension and no problems in other dimensions (the EQ-5D-5L profile “11121”) was the most common profile. The PCHC analysis suggested that at 3/12 months follow-ups, the EQ-5D-5L profiles improved for 46.9%/50.4% of participants, worsened for 19.5%/20.1%, showed mixed changes for 19.7%/18.7% and remained unchanged for 13.9%/10.9%.

**Conclusion:**

In this single-arm registry study, about half of participants reported improved EQ-5D-5L profiles at 3 and 12 months following digital education and exercise therapy, while one-fifth reported worsening and another one-fifth showed mixed changes, indicating heterogeneous HRQoL trajectories and the need for tailored participant support.

**Supplementary Information:**

The online version contains supplementary material available at https://doi.org/10.1186/s12955-026-02634-5.

## Introduction

Osteoarthritis (OA) is the most common form of arthritis, and a leading cause of pain and disability [1]. Pain and physical functional limitations associated with OA lead to significant impairments in health-related quality of life (HRQoL), often resulting in decreased physical activity, social isolation, and psychological distress [2–4]. HRQoL is a subjective assessment of an individual’s physical, mental, and social domains of life [5]. With no cure for OA and its chronic nature, HRQoL constitutes an important aspect of OA management and is widely used to evaluate the effectiveness of OA treatments [3].

Patient education, exercise and weight control are recommended as core first-line nonpharmacologic treatment for OA [6]. Recent years have witnessed a remarkable rise in the digital delivery of these first-line treatments [7]. Previous studies have suggested improvements in pain, physical functions and HRQoL following participation in digital education and exercise programs [8, 9]. However, previous evidence is mainly from randomised controlled trials with small sample sizes and short follow-ups including predominantly individuals with knee OA [8, 9]. As OA is a lifelong condition and the benefits of education and exercise therapy depend on sustained self-management after the supervised part of a program ends, an adequate follow-up should extend beyond program completion to capture whether early gains in HRQoL are maintained rather than only the immediate post-treatment response. Moreover, while HRQoL can be measured using generic or disease-specific questionnaires [10], previous studies mainly relied on disease-specific HRQoL measures. Therefore, there is little real-world data on the changes in HRQoL, measured using a generic measure, following participation in digital education and exercise therapy for knee and hip OA.

The EQ-5D is one of the most widely used generic HRQoL measures across populations and health conditions [11]. It is also the most collected HRQoL measure by the National Quality Registries in Sweden [12]. EQ-5D data are generally reported and analysed as a single summary score derived from a population-based value set [13]. Although useful to inform economic evaluations, reliance on a single index score entails a loss of clinically relevant information, particularly regarding which specific health dimensions are affected by disease or intervention [13, 14]. The reference value sets used to compute the summary scores are gathered from a sample of the general population or patient groups who may have different preferences and priorities than the cohort under study. Moreover, the summary scores derived using different reference value sets are not directly comparable [13, 15]. To address these limitations, especially when assessing HRQoL is the main interest, one can analyse EQ-5D profile data to provide a more detailed and comprehensive information on HRQoL in a sample [13, 15]. Profile analysis should, however, be regarded as complementary to rather than a substitute for analysis based on value sets, especially given that some profile analysis methods themselves rely on value sets to rank health states [15]. In the present study, we explored longitudinal changes in HRQoL using the EQ-5D-5L profile data up to 1 year following participation in a digital education and exercise program for knee and hip OA in Sweden.

## Methods

### Data source

We used data from the Joint Academy^®^ registry, a digital platform for delivering education and exercise therapy for knee and hip OA in Sweden [16]. The digital program is described in detail elsewhere [16, 17]. In short, participants receive video lectures on OA, physical activity, and self-management as well as individualized exercises adjusted according to their progression in the program through a smartphone application. They also receive regular supervision from their physiotherapist via telephone or video calls, with the option of asynchronous chat communication during participation in the program. Although participants may continue in the program if they wish, the core content and basic package are delivered within the first 12 weeks.

### Participants

**Fig. 1:**
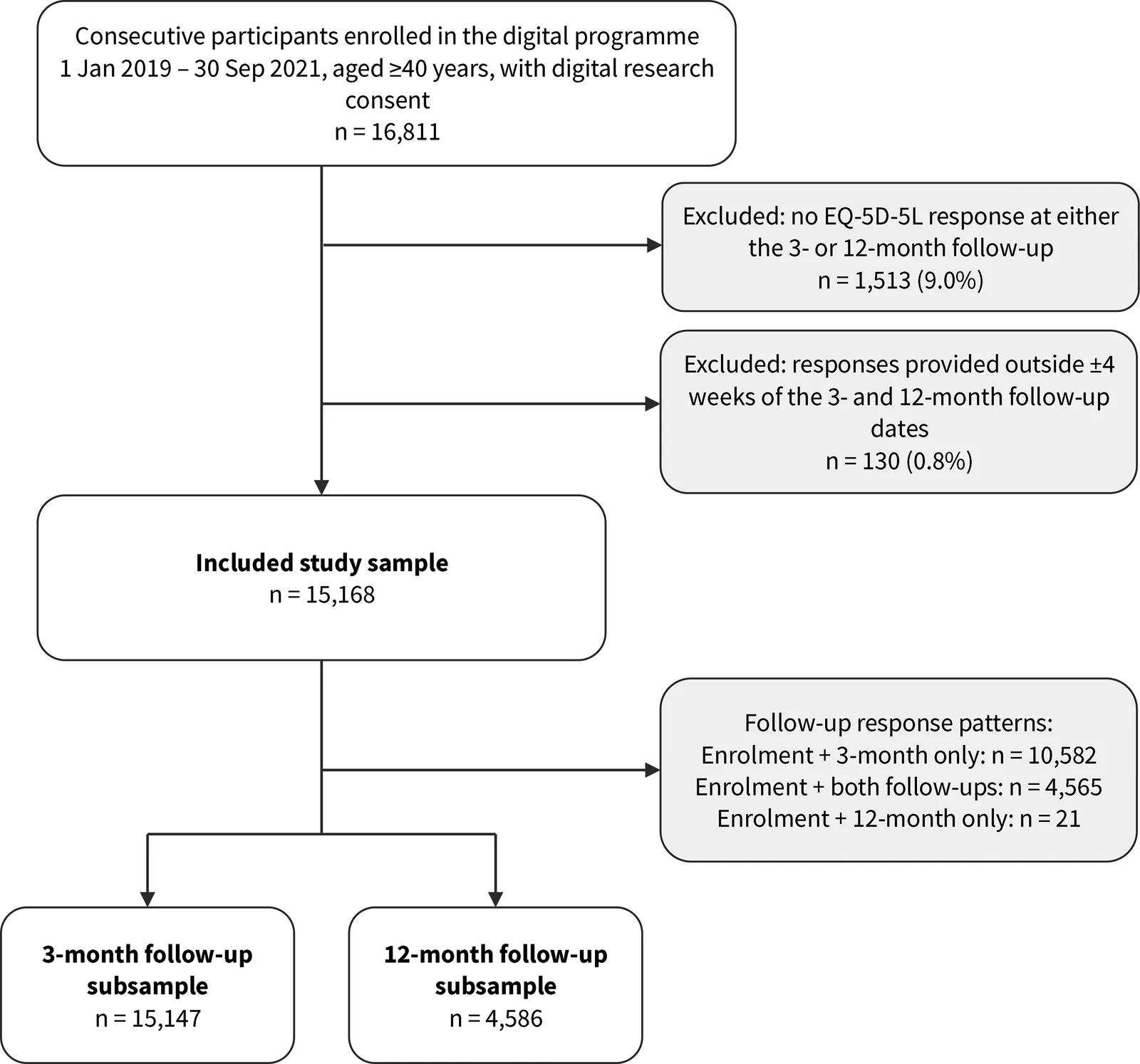
Flowchart of sample selection

All consecutive participants aged 40 + years enrolled in the program between January 1st, 2019, and September 30, 2021, who provided their digital consent for research at enrolment were included (*n* = 16,811). Among these, we excluded 1513 (9.0%) participants with no response to the EQ-5D-5L at any of 3- or 12-month follow-ups. Additionally, 130 (0.8%) of participants provided responses outside ± 4 weeks of the 3- and 12-month follow-up dates and were excluded (Fig. 1).

### EQ-5D-5L

The EQ-5D-5L is a reliable and valid generic HRQoL measure for use among people with OA [18, 19]. The EQ-5D-5L covers five important dimensions of health: mobility, selfcare, usual activities, pain/discomfort, and anxiety/depression. While in the original EQ-5D (i.e., EQ-5D-3L) each dimension had three response options (levels), this rose to 5 in the EQ-5D-5L to improve precision and responsiveness [20, 21]. These 5 levels are: (1) no problems, (2) slight problems, (3) moderate problems, (4) severe problems, and (5) unable to/extreme problems. Combining dimensions and levels results in 3125 (5^5) unique EQ-5D-5L profiles. A profile can be described as a 5-digit number representing the level of problems in each dimension [15]. For instance, an EQ-5D-5L profile of “12345” indicates “no problems” in mobility, “slight problems” in selfcare, “moderate problems” in usual activities, “severe problems” in pain/discomfort, and “unable to/extreme problems” in anxiety/depression. An EQ-5D-5L profile can be summarized as a single score (known as the EQ-5D index score) anchored at 1 (full health) and 0 (a state equivalent to death) using a reference value set with values less than zero representing profiles considered to be worse than dead [15]. Within the digital program, EQ-5D-5L responses are collected by design at enrolment and at 3, 6, 9, and 12 months thereafter. In the present study, we analysed only the earliest and latest follow-ups (3 and 12 months).

### Data analysis

General characteristics of participants at enrolment were reported as mean and standard deviation (SD) and numbers/proportions. We reported the mean EQ-5D-5L index score using the Swedish value set ranging from − 0.314 (worst health state) to 1 (full health) [22]. We also reported the numbers and proportions of the responses to different levels of each EQ-5D-5L dimension at enrolment and 3- and 12-month follow-ups.

To investigate changes in each dimension from enrolment, we divided changes in each dimension into four groups: (1) improved, (2) worsened, (3) no change, and (4) no problems (i.e. reporting no problems at both enrolment and follow-up). To identify the dimensions most and least influenced by the disease and intervention context, we computed the probability of superiority as the sum of the number of improved and half of the number of no change and no problems within the same dimension divided by the total number of responses [21]. This is the probability that within a randomly selected pair of responses, the response obtained at follow-up is better than the response at enrolment. It ranges from 0 to 1 with values higher (lower) than 0.5 suggesting higher improvement (deterioration).

In analysing the EQ-5D-5L profiles, we reported the 10 most common profiles in each data point (i.e., enrolment, 3- and 12-month follow-ups). We then used the Pareto Classification of Health Change (PCHC) to assess changes in the EQ-5D-5L profiles as follows [13]:

- Improved: improvement in at least one dimension with no deterioration in any other dimension.
- Worsened: deterioration in at least one dimension with no improvement in any other dimension.
- No change: the same EQ-5D-5L profile.
- Mixed changes: improved in at least one dimension but worsened in at least one other dimension.

We computed 95% confidence intervals (CIs) for these proportions using the Goodman’s simultaneous confidence interval for multinomial proportions [23]. We also repeated the PCHC analysis stratified by baseline EQ-5D-5L index score to explore the potential influence of ceiling and floor effects and of regression to the mean. We additionally visualized the changes in the EQ-5D-5L profiles using the Health Profile Grid (HPG) which plots the profiles between enrolment and each follow-up [13]. This approach relies on the EQ-5D-5L profiles being ranked from best to worst. We ranked the EQ-5D-5L profiles using the Swedish EQ-5D-5L value set [22]. In a HPG plot, the 45-degree line represents “no change” with points above (below) the line indicated that the profile improved (worsened) with further distance from the line suggesting greater improvements (deteriorations). We then computed the probability of superiority and corresponding 95% CI using 5000 bootstrap replications based on the ranks of the EQ-5D-5L profile between enrolment and follow-ups. It should be noted that because both HPG and the probability of superiority depend on an EQ-5D-5L value set, they share the value set limitations outlined earlier. All data management and statistical analyses were conducted in Stata v.18.

## Results

A total of 15,168 individuals with mean (SD) age 64.3 (8.8) and 75.3% females were included. Of included individuals, 10,582 individuals provided responses at enrolment and only 3 months follow-up, 4565 individuals at enrolment and both follow-ups, and 21 individuals at enrolment and only 12 months follow-up, resulting in subsamples of 15,147 and 4586 individuals for 3- and 12-month follow-ups, respectively (Table 1). Compared with those included, individuals excluded were slightly older with a higher proportion of males, hip OA and comorbidities and a lower Swedish EQ-5D-5L index score at enrolment. Moreover, individuals participating in the program at 12-months (*n* = 4586) were comparable to those who didn’t participate at that time point (*n* = 10582) (Table S1 in the supplement).

**Table 1:** Baseline characteristics of included and excluded participants

	Included		Excluded
	3-month follow-up	12-month follow-up	
N	15,147	4586	1643
Age, mean (SD)	64.3 (8.8)	64.4 (8.5)	65.8 (9.8)
Female, n (%)	11,401 (75.3)	3470 (75.7)	1188 (72.3)
Knee osteoarthritis, n (%)	9060 (59.8)	2807 (61.2)	882 (53.7)
Comorbidity, n (%)^a^	3938 (26.0)	1205 (26.3)	545 (33.2)
Educational attainment, n (%)			
Less than high school	1323 (8.7)	381 (8.3)	157 (9.6)
High school	5487 (36.2)	1551 (33.8)	570 (34.7)
College/university	8337 (55.0)	2654 (57.9)	916 (55.8)
Body mass index, mean (SD)	27.1 (4.7)	27.0 (4.7)	27.2 (5.0)
NRS Pain (0 to 10), mean (SD)	5.1 (1.9)	5.1 (1.9)	5.1 (2.1)
Swedish EQ-5D-5L index (-0.314 to 1.0)	0.847 (0.166)	0.847 (0.167)	0.819 (0.196)

SD: standard deviation; NRS: Numeric rating scale

^a^ Presence of any of following self-reported doctor diagnosed comorbidities: diabetes, lung diseases, balance troubles, rheumatoid arthritis or cardiovascular diseases

### The EQ-5D-5L dimensions

**Fig. 2:**
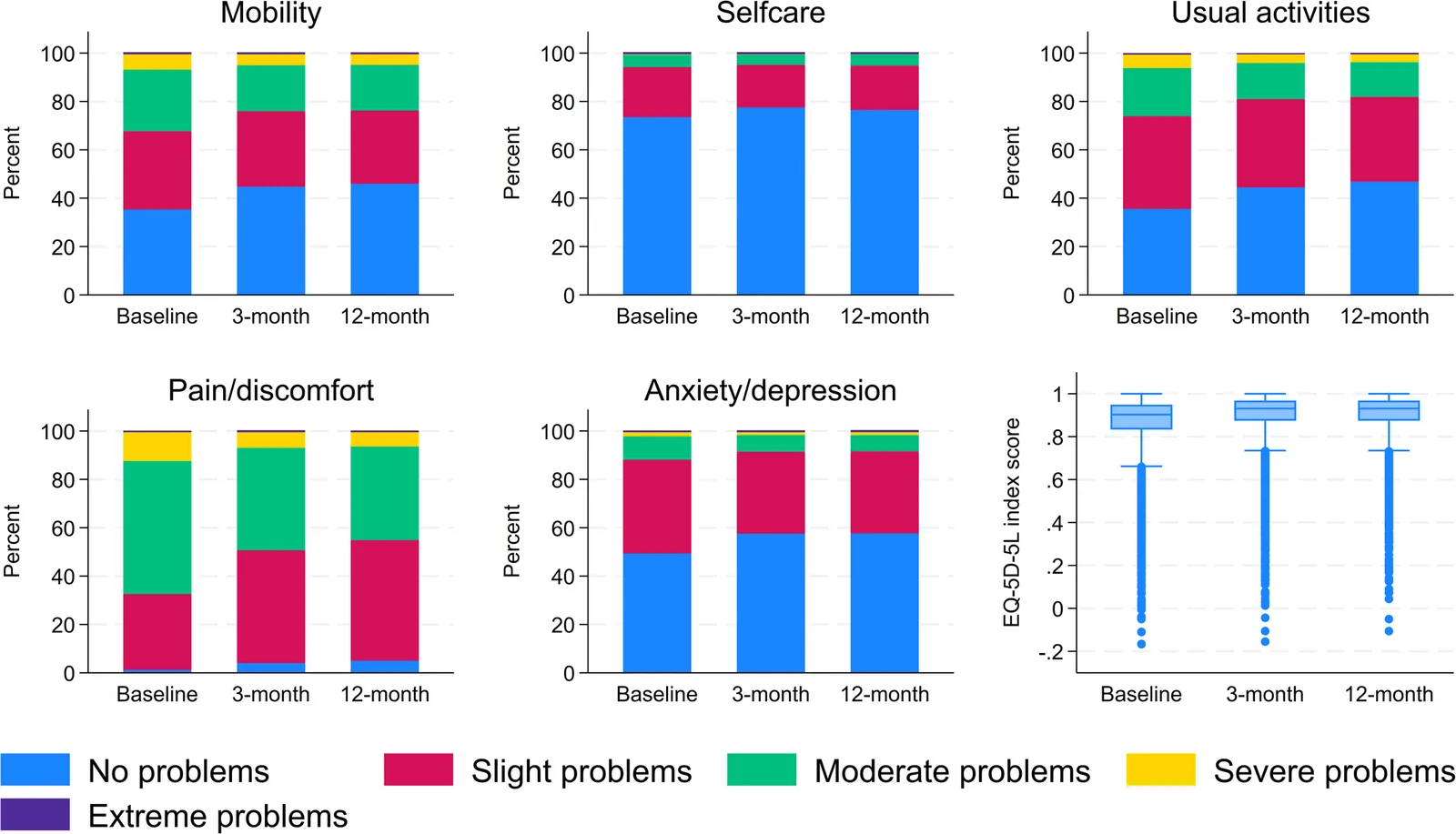
Distributions of responses to each EQ-5D-5L dimensions and the Swedish EQ-5D-5L index score at enrolment and follow-ups

In both follow-ups, the percentage of individuals with problems in 0–2 dimensions increased, while the percentage of those with problems in 3–5 dimensions decreased from enrolment (Table S2 in the supplement). While about 14% of the participants reported problems only in the pain/discomfort dimension, the proportion reporting problems only in a single dimension was less than 0.3% for other dimensions. The percentages of persons reporting moderate to extreme problems at enrolment ranged from around 6% for selfcare to 68% for pain/discomfort in both subsamples (Fig. 2). At enrolment, “no problems” was the most common response level for mobility, selfcare, and anxiety/depression, whereas “slight problems” was the most common level for usual activities and “moderate problems” for pain/discomfort. At 3- and 12-month follow-ups, the proportions of participants reporting moderate to extreme problems dropped compared to enrolment for all dimensions and “no problems” became the most common response for all dimensions except pain/discomfort, where “slight problems” was the most common. The proportion of individuals reporting extreme problems (level 5) in at least one dimension decreased from 1.2/1.3% at enrolment to 0.9/0.8% at the 3-/12-month follow-ups.

**Fig. 3:**
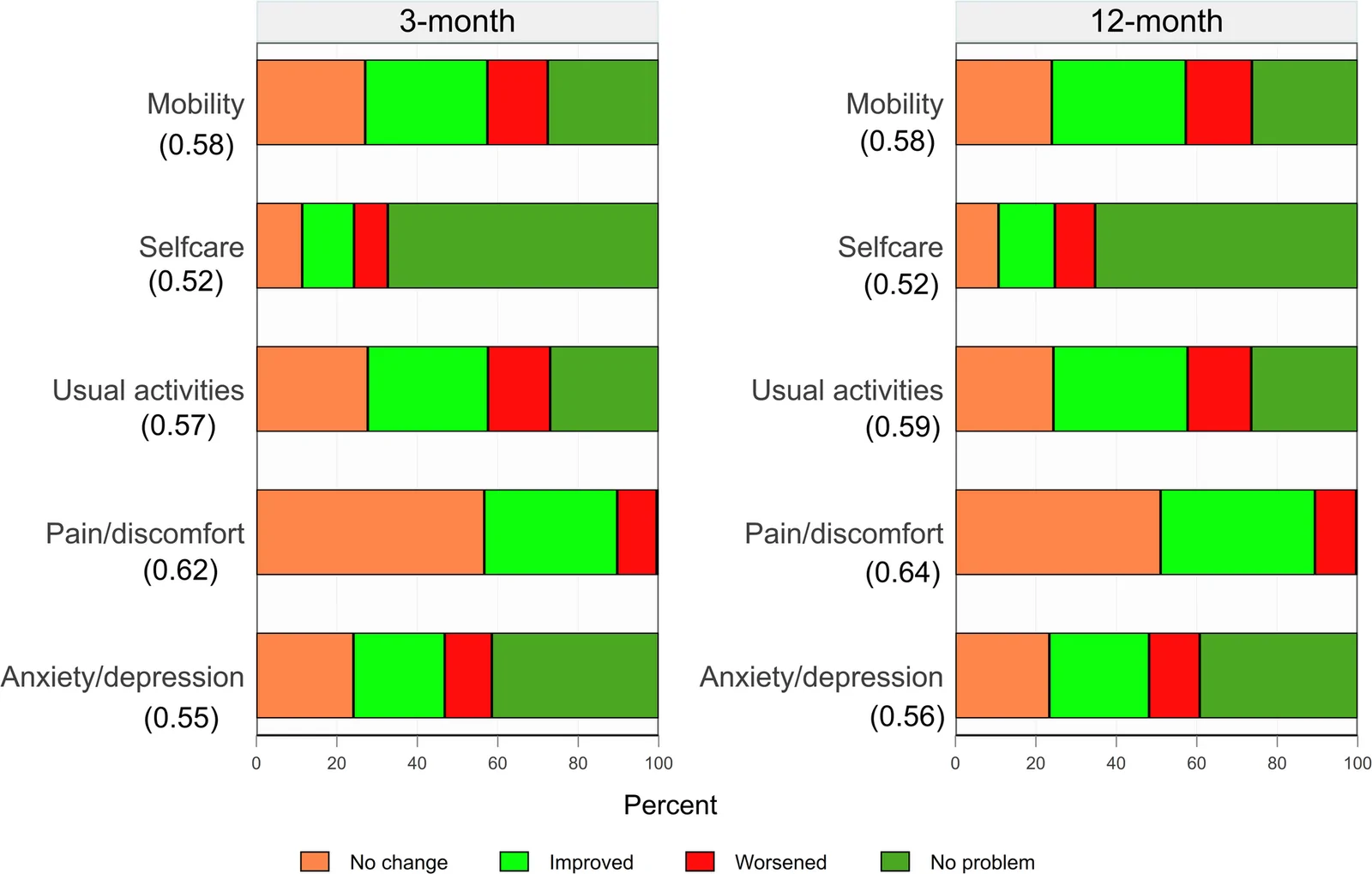
Patterns of changes in the EQ-5D-5L dimensions from enrolment to 3- and 12-month follow-ups. Values in parentheses show the probability of superiority for each dimension

The largest proportions of “improvement” were reported for pain/discomfort at both follow-ups while the largest proportions of “worsening” were seen for usual activities in 3-month and for mobility in 12-month (Fig. 3). Among individuals reporting some levels of pain/discomfort at enrolment, about 57% and 51% reported the same levels at 3- and 12-month follow-ups, respectively (i.e. no change in pain/discomfort severity). The probability of superiority showed that for all dimensions, more patients improved than worsened with selfcare and pain/discomfort as the least and most improved dimensions, respectively.

### The EQ-5D-5L profiles

At enrolment, a total of 561/377 out of 3125 possible EQ-5D-5L profiles were observed in the 3-/12-month subsamples with slight or moderate problems in pain/discomfort dimension and no problems in any other dimension (i.e., profiles “11121” and “11131”) being the most common profiles. The profiles “11121” and “11122” were the first and second most common profile at both follow-ups (the top 10 EQ-5D-5L profiles at enrolment and follow-ups are reported in Table S3 in the supplement). While “no problems” at any dimension (the EQ-5D-5L profile “11111”) accounted for 0.7%/0.6% of the responses at enrolment in 3-/12-month subsamples, it rose to 3.1%/3.8% of the responses at 3- and 12-month follow-ups.

**Fig. 4:**
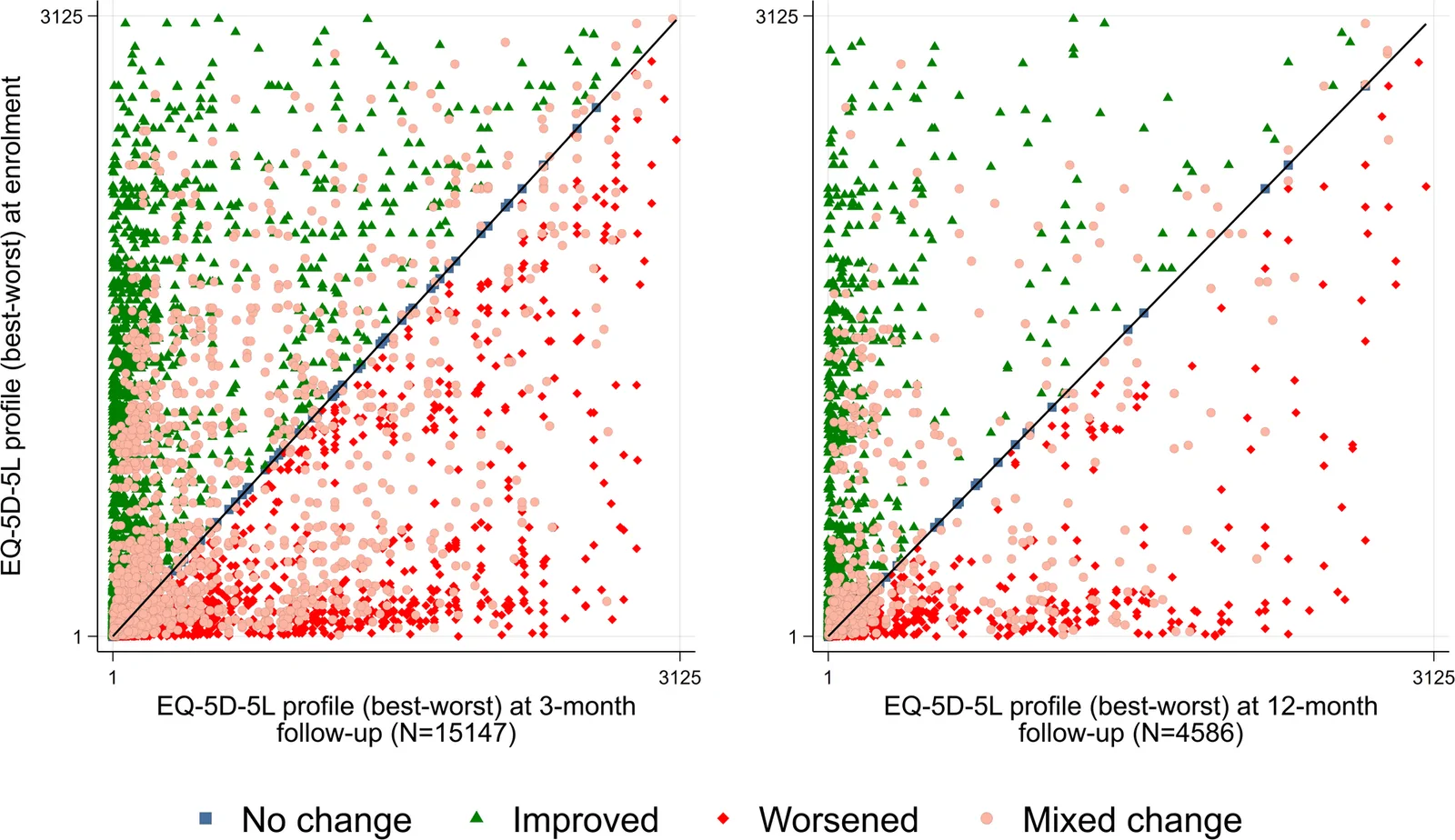
Comparison of EQ-5D-5L profiles at enrolment and 3-/12-month follow-ups. Profiles were ranked using the Swedish value set

The PCHC analysis suggested that the EQ-5D-5L profiles improved for 46.9% (95% CI: 45.9, 47.9) and 50.4% (48.5, 52.2) of participants at 3- and 12-month after enrolment, respectively (Table 2). On the other hand, it worsened for about 20% of participants in both follow-ups. In the HPG plots (Fig. 4), 8838 (58.3%) and 2799 (61.0%) participants were above the diagonal line at 3- and 12-month follow-ups, respectively, while 4098 (27.1%) and 1259 (27.5%) were below the line at these follow-ups. The probability of superiority suggested that there were 65.6% (95% CI 64.9, 66.4) and 66.8% (65.5, 68.1) chance that a randomly selected EQ-5D-5L profile at 3- and 12-month follow-ups, respectively, were better than a randomly selected profile at enrolment.

**Table 2:** Changes in the EQ-5D-5L profile according to the Pareto Classification of Health Change

Change pattern	3-month subsample (*n* = 15147)	12-month subsample (*n* = 4586)
No change, n (%: 95% CI)	2104 (13.9: 13.2, 14.6)	498 (10.9: 9.8, 12.1)
Improved, n (%, 95% CI)	7100 (46.9: 45.9, 47.9)	2311 (50.4: 48.5, 52.2)
Worsened, n (%, 95% CI)	2957 (19.5: 18.7, 20.3)	920 (20.1: 18.6, 21.6)
Mixed change, n (%, 95% CI)	2986 (19.7: 18.9, 20.5)	857 (18.7: 17.3, 20.2)

Among participants with an improved profile (*n* = 7100 at 3-month and *n* = 2311 at 12-month), 36.6% and 33.5% of participants, respectively, reported improvement in only one dimension at 3- and 12-month follow-up (Table S4 in supplement). Of these, improvement in pain/discomfort with no changes in other dimensions was the most common pattern in both 3- and 12-month follow-ups. Moreover, simultaneous improvements in mobility, usual activities and pain/discomfort dimensions with no change in two other dimensions was the fourth and second most common pattern at 3- and 12-month follow-ups, respectively. In the whole sample, 1.4% and 2.0% reported improvements in all five dimensions at 3- and 12-month follow-ups, respectively.

Among those whose profile worsened (*n* = 2957 at 3-month and *n* = 920 at 12-month), 50.3% and 41.1% reported worsening in one dimension only at 3- and 12-month follow-ups, respectively (Table S5 in supplement). Of these, worsening in only mobility or in only usual activities with no change in other dimensions were the patterns with the largest frequencies.

For those with mixed changes in their EQ-5D-5L profiles, the percentages of participants with a higher number of improved than worsened dimensions were greater than the opposite change at both follow-ups (36.4% vs. 19.5% at 3-month and 32.0% vs. 19.7% at 12-month subsamples, Table S6 in supplement).

The PCHC analysis stratified by EQ-5D-5L index score at enrolment revealed that those with better HRQoL at enrolment were more (less) likely to report a worsening or no change (improvement) in their EQ-5D-5L profile signalling the potential contributions of ceiling/floor artefact and/or regression to the mean (Table S7 in supplement).

From enrolment, the Swedish EQ-5D-5L index score improved on average by 0.039 (95% CI: 0.037, 0.041) at 3-month and by 0.044 (0.039, 0.048) at 12-month.

## Discussion

In this large registry-based longitudinal study, we found notable improvements in HRQoL measured using EQ-5D-5L, with nearly half of participants reporting improvements in their EQ-5D-5L profiles up to one year after the digital program while around 20% reported deteriorations. Selfcare and pain/discomfort were the least and most affected EQ-5D-5L dimensions at enrolment, respectively, which resulted in these dimensions seeing the least and most improvements following the digital treatment.

The order of EQ-5D-5L dimensions from the least to most frequent with moderate to extreme problems was selfcare, anxiety/depression, usual activities, mobility and pain/discomfort. The same ordering has been reported among individuals with knee or hip OA participating in face-to-face education and exercise program and those undergoing total knee and hip arthroplasties in Sweden [24, 25] and patients with knee OA prior to total knee arthroplasty in China and Canada [26, 27]. In the study conducted in China [26], the most common EQ-5D-5L profile was “11121”, as in the present study. However, contrary to our findings, the EQ-5D-5L profile “11111” (no problem at any dimension) was the second most frequent profile in China and only 4 out of the 10 most frequent EQ-5D-5L profiles at enrolment in the present study overlapped with those in the Chinese study [26]. These findings highlight that while EQ-5D-5L responses generally present a consistent pattern across OA populations, the detailed distribution of severity level varies reflecting potential differences in patient-mix and care context as well as cross-cultural effects.

Interestingly, the order of dimensions with most frequent moderate to extreme problems in the present study was the same as in the Swedish general population [28]. However, proportions were higher for all dimensions in the present study, with the largest absolute and relative differences seen for pain/discomfort dimension at enrolment (67.4% vs. 28.1%) highlighting significant contribution of OA-related pain to HRQoL [28]. As expected, the proportion of the individuals reporting no problem at any dimension (the EQ-5D-5L profile “11111”) was lower in the present study compared with the Swedish general population (0.7% at enrolment to about 4.0% at follow-ups vs. 24.1%) [28]. Surprisingly, a lower proportion of individuals reported extreme problems (level 5) in at least one EQ-5D-5L dimension in our sample compared with the Swedish general population (1.2% vs. 2.9%) [28]. This may partially be due to difference in administering EQ-5D-5L questionnaire (digital vs. postal survey), and sociodemographic differences (e.g. 75.3% vs. 52.6% females, 8.7% vs. 23.2% with low educational attainment) [28]. It may also suggest a selection issue whereas most impaired OA patients with severe disability might be under-represented among participants of the digital program. Moreover, consistent with “response shift” theory [29], people living with OA may recalibrate what they consider “extreme,” leading them to select “severe problems” (level 4) rather than “extreme problems” (level 5) even when the level of impairment is high.

Around half of the participants in the digital program reported improvement in their EQ-5D-5L profile following participation in the digital program. Across single dimensions, improvements ranged from about 13%/14% for selfcare to 33%/38% for pain/discomfort at 3/12 months follow-ups. These improvement in HRQoL measured with EQ-5D-5L following education and exercise therapy are consistent with previous studies [30–32]. The magnitude of change in the EQ-5D-5L index score (0.04) in the present study is almost equivalent to the estimated minimal important change [33, 34] and comparable to those reported following education and exercise programs in Denmark and Australia [31, 32]. These improvements are smaller than those reported among people undergoing knee or hip arthroplasty [35, 36]. For instance, the proportion of the full health profile (“11111”) increased from 0.06% pre-operation to 28.6% at 1-year post-operation and EQ-5D-5L index score improved by 0.22 among individuals undergoing hip arthroplasty in Sweden [35]. The corresponding changes in the present study were from 0.6% to 3.8% with a ≈ 0.04 improvements in the EQ-5D-5L index score. This may be due to more severe HRQoL impairment pre-operation among those undergoing knee or hip arthroplasties, and hence more room for improvement following intervention (e.g. the baseline mean EQ-5D-5L index score of 0.85 in the present study vs. 0.66 among those undergoing hip arthroplasty [35]). While improvements reported in the present study are encouraging and support the use of digital education and exercise programs in OA population, around one third of the participants reported worsening or no change in their EQ-5D-5L profile. Looking specifically at the pain/discomfort dimension, the main driver of HRQoL impairment in this population, over 50% reported no change and 10% reported worsening. These results highlight the need for additional support in the digital program and adding alternative treatments to the program.

Several limitations of the current study should be acknowledged. Without a control group, the observed improvements in HRQoL cannot be fully attributed to the digital intervention as these improvements could reflect other factors such as placebo effects, natural history of the disease, concurrent interventions, or response shift. For instance, enrolment may coincide a peak in symptoms, so that the subsequent improvement reflects regression to the mean and the fluctuating course of the disease rather than any actual effect of the program. Participation in the digital intervention is voluntary and requires access to, and familiarity with, digital technologies and Swedish language proficiency. These issues may result in possible differences between persons who participated in the program from the broader OA population limiting the generalizability of the findings. We used the EQ-5D-5L to assess HRQoL which showed ceiling effects at dimension level (i.e. for all dimensions except pain/discomfort more than 15% of participants reported “no problems” at enrolment). Moreover, a previous item response theory analysis suggested that while EQ-5D-5L has acceptable psychometric properties among participants of the same digital intervention, it targeted, on average, a poorer HRQoL than experienced by the participants [18]. This can reduce the responsiveness of the EQ-5D-5L to detect improvements among those with mild HRQoL impairment at baseline. Finally, while our 12-month follow-up is longer than in most previous studies of digital first-line OA interventions, it remains short relative to the lifelong course of OA. Whether the observed changes in the EQ-5D-5L profile persist beyond one year, and how they evolve as program engagement declines are subjects for future studies.

## Conclusion

In this single-arm evaluation of a digital education and exercise programme for knee and hip OA, consistent with typical HRQoL patterns in OA, pain/discomfort was the main driver of HRQoL impairment. While pain/discomfort had the greatest improvements among the EQ-5D-5L dimensions, around 60% of participants reported no change (50%) or worsening (10%) in pain/discomfort. Moreover, while nearly half of the participants reported improvements in their EQ-5D-5L profile, these important gains were partially offset by one in five participants reporting worsening in their EQ-5D-5L profile. These results suggest a need for additional tailored support, adjunct non-surgical options, and/or closer monitoring of participants in digital treatment.

## Supplementary Information

Below is the link to the electronic supplementary material.

Supplementary Material 1

## Data Availability

The data that support the findings of this study are available from Joint Academy^®^, but restrictions apply to the availability of these data, which were used under ethical permission for the current study, and so are not publicly available. Data may be made available through the corresponding author upon reasonable request and with permission of Joint Academy^®^.

## Abbreviations

HPG: Health Profile Grid
HRQoL: Health-related quality of life
OA: Osteoarthritis
PCHC: Pareto Classification of Health Change
