# Supplementary material for: Changes in health-related quality of life: a longitudinal registry study among over 15,000 participants of a digital education and exercise therapy for knee and hip osteoarthritis using EQ-5D-5L
Source: Health Qual Life Outcomes. 2026 Sep 19;24:121. doi: 10.1186/s12955-026-02634-5 (PMC13591732; doi:10.1186/s12955-026-02634-5)
Supplement: Supplementary file 1 [file 12955_2026_2634_MOESM1_ESM.pdf]

Table S1. Baseline characteristics of 12-month responders vs non-responders.

	Responders	Non-responders
N	4586	10582
Age, mean (SD)	64.4 (8.5)	64.3 (9.0)
Female, n (%)	3470 (75.7)	7946 (75.1)
Knee osteoarthritis, n (%)	2807 (61.2)	6262 (59.2)
Comorbidity, n (%)[a]	1205 (26.3)	2735 (25.9)
Educational attainment, n (%)		
Less than high school	381 (8.3)	943 (8.9)
High school	1551 (33.8)	3942 (37.3)
College/university	2654 (57.9)	5697 (53.8)
Body mass index, mean (SD)	27.0 (4.7)	27.2 (4.7)
NRS Pain (0 to 10), mean (SD)	5.1 (1.9)	5.1 (1.9)
Swedish EQ-5D-5L index (-0.314 to 1.0)	0.847 (0.167)	0.847 (0.166)

SD: standard deviation; NRS: Numeric rating scale

[a] Presence of any of following self-reported doctor diagnosed comorbidities: diabetes, lung diseases, balance troubles, rheumatoid arthritis or cardiovascular diseases.

Table S2. Number (%) of persons with problems in the EQ-5D-5L dimensions.

# of dimensions with problems	3-month subsample (n=15147)		12-month subsample (n=4586)	
	Enrolment	3-month follow-up	Enrolment	12-month follow-up
0	108 (0.7)	462 (3.1)	28 (0.6)	175 (3.8)
1	2249 (14.8)	3188 (21.0)	661 (14.4)	1028 (22.4)
2	2990 (19.7)	3288 (21.7)	916 (20.0)	984 (21.5)
3	3604 (23.8)	3317 (21.9)	1071 (23.4)	911 (19.9)
4	3832 (25.3)	3014 (19.9)	1166 (25.4)	879 (19.2)
5	2364 (15.6)	1878 (12.4)	744 (16.2)	609 (13.3)

Table S3. Ten most common EQ-5D-5L health profiles at enrolment and follow ups.

Rank	3-month subsample		12-month subsample	
	Enrolment	3-month	Enrolment	12-month
1	11121 (9.6)	11121 (16.5)	11121 (9.1)	11121 (19.0)
2	11131 (4.7)	11122 (4.9)	11131 (4.7)	11122 (5.5)
3	21231 (3.9)	21121 (4.2)	21231 (4.0)	21121 (4.3)
4	11122 (3.4)	11221 (3.9)	11122 (3.8)	11111 (3.8)
5	21232 (3.3)	11131 (3.7)	21232 (3.3)	11221 (3.4)
6	21121 (2.7)	21221 (3.6)	11221 (2.6)	21221 (3.4)
7	11221 (2.5)	21231 (3.1)	21131 (2.6)	21231 (2.7)
8	21131 (2.5)	11111 (3.1)	31332 (2.6)	11131 (2.5)
9	11132 (2.4)	21232 (2.7)	21121 (2.6)	21232 (2.4)
10	21221 (2.4)	21222 (2.3)	11132 (2.4)	21131 2.0)

1: “No problems”; 2: "Slight problems"; 3: "Moderate problems"; 4: "Severe problems"; 5: "Extreme problems". Dimensions are displayed in following order: “mobility”, “self-care” “usual activities”, "pain/discomfort” and “anxiety/depression”.

Table S4. Pattern of changes in the EQ-5D-5L dimensions among those who experienced improvements in the EQ-5D-5L profile from enrolment.

	3-month subsample		12-month subsample	
	Number	% from total sample	Number	% from total sample
One dimension	2598	17.2	773	16.9
MO	601	4.0	158	3.4
SC	163	1.1	52	1.1
UA	569	3.8	163	3.6
PD	825	5.4	275	6.0
AD	440	2.9	125	2.7
Two dimensions	2163	14.3	633	13.8
MO + SC	71	0.5	15	0.3
MO + UA	396	2.6	101	2.2
MO + PD	413	2.7	132	2.9
MO + AD	194	1.3	49	1.1
SC + UA	111	0.7	30	0.7
SC + PD	71	0.5	29	0.6
SC + AD	63	0.4	21	0.5
UA + PD	383	2.5	106	2.3
UA + AD	189	1.2	52	1.1
PD + AD	272	1.8	98	2.1
Three dimensions	1405	9.3	500	10.9
MO + SC + UA	91	0.6	27	0.6
MO + SC + PD	82	0.5	22	0.5
MO + SC + AD	41	0.3	11	0.2
MO + UA + PD	512	3.4	216	4.7
MO + UA + AD	168	1.1	41	0.9
MO + PD + AD	192	1.3	65	1.4
SC + UA + PD	75	0.5	25	0.5
SC + UA + AD	54	0.4	17	0.4
SC + PD + AD	35	0.2	12	0.3
UA + PD + AD	155	1.0	64	1.4
Four dimensions	725	4.8	311	6.8
MO + SC + UA + PD	192	1.3	71	1.5
MO + SC + UA + AD	74	0.5	22	0.5
MO + SC + PD + AD	55	0.4	20	0.4
MO + UA + PD + AD	342	2.3	180	3.9
SC + UA + PD + AD	62	0.4	18	0.4
Five dimensions	209	1.4	94	2.0

MO: Mobility, SC: Selfcare, UA: Usual activities, PA: Pain/discomfort, AD: Anxiety/depression.

Table S5. Pattern of changes in the EQ-5D-5L dimensions among those who experienced worsening in the EQ-5D-5L profile from enrolment.

	3-month subsample		12-month subsample	
	Number	% from total sample	Number	% from total sample
One dimension	1486	9.8	378	8.2
MO	389	2.6	115	2.5
SC	141	0.9	39	0.9
UA	399	2.6	88	1.9
PD	284	1.9	60	1.3
AD	273	1.8	76	1.7
Two dimensions	865	5.7	269	5.9
MO + SC	48	0.3	30	0.7
MO + UA	230	1.5	65	1.4
MO + PD	101	0.7	33	0.7
MO + AD	76	0.5	24	0.5
SC + UA	85	0.6	29	0.6
SC + PD	19	0.1	6	0.1
SC + AD	49	0.3	14	0.3
UA + PD	94	0.6	33	0.7
UA + AD	99	0.7	21	0.5
PD + AD	64	0.4	14	0.3
Three dimensions	409	2.7	171	3.7
MO + SC + UA	62	0.4	27	0.6
MO + SC + PD	13	0.1	5	0.1
MO + SC + AD	23	0.2	11	0.2
MO + UA + PD	57	0.4	50	1.1
MO + UA + AD	103	0.7	26	0.6
MO + PD + AD	36	0.2	16	0.3
SC + UA + PD	27	0.2	9	0.2
SC + UA + AD	30	0.2	12	0.3
SC + PD + AD	19	0.1	4	0.1
UA + PD + AD	39	0.3	11	0.2
Four dimensions	169	1.1	72	1.6
MO + SC + UA + PD	47	0.3	28	0.6
MO + SC + UA + AD	31	0.2	21	0.5
MO + SC + PD + AD	19	0.1	4	0.1
MO + UA + PD + AD	49	0.3	17	0.4
SC + UA + PD + AD	23	0.2	2	0.0
Five dimensions	28	0.2	30	0.7

MO: Mobility, SC: Selfcare, UA: Usual activities, PA: Pain/discomfort, AD: Anxiety/depression.

Table S6. Pattern of changes in the EQ-5D-5L dimensions among those with mixed change in the EQ-5D-5L profile from enrolment.

		From enrolment to 3-month after enrolment					From enrolment to 12-month after enrolment			
# of dimensions	***Improved***	1	2	3	4		1	2	3	4
Worsened										
**1**	***Same***					***Same***				
	0				49	0				19
	1			248		1			95	
	2		644			2		196		
	3	1324				3	348			
**2**										
	0			16		0			2	
	1		118			1		30		
	2	431				2	111			
**3**										
	0		15			0		8		
	1	122				1	41			
**4**										
	0	19				0	7			

Table S7. Changes in the EQ-5D-5L profile according to the Pareto Classification of Health Change, stratified by quartiles of the of Swedish EQ-5D-5L score at enrolment.

Change pattern	3-month subsample (n=15147)				12-month subsample (n=4586)			
	Q1 (Lowest)	Q2	Q3	Q4 (Highest)	Q1 (Lowest)	Q2	Q3	Q4 (Highest)
No change, n (%)	133 (3.5)	346 (8.4)	353 (10.2)	1272 (34.0)	38 (3.3)	55 (4.7)	81 (7.1)	324 (28.8)
Improved, n (%)	2260 (58.9)	2235 (54.3)	1615 (46.7)	990 (26.5)	731 (63.3)	665 (56.9)	573 (50.4)	342 (30.4)
Worsened, n (%)	401 (10.5)	720 (17.5)	724 (20.9)	1112 (29.7)	114 (9.9)	212 (18.1)	238 (21.0)	356 (31.6)
Mixed change, n (%)	1042 (27.2)	812 (19.7)	766 (22.2)	366 (9.8)	272 (23.6)	237 (20.3)	244 (21.5)	104 (9.2)
